# Platelet mitochondrial membrane depolarization reflects disease severity in patients with sepsis and correlates with clinical outcome

**DOI:** 10.1186/cc13724

**Published:** 2014-02-12

**Authors:** Katharina Gründler, Matthias Angstwurm, Robert Hilge, Philipp Baumann, Thorsten Annecke, Alexander Crispin, Hae-Young Sohn, Steffen Massberg, Bjoern F Kraemer

**Affiliations:** 1 Walter Brendel Zentrum für Experimentelle Medizin, LMU München, München, Germany; 2 Medizinische Klinik und Poliklinik IV, Klinikum der Universität München, München, Germany; 3 Klinik für Anästhesiologie, Klinikum der Universität München, München, Germany; 4 Institut für Medizinische Informationsverarbeitung, Biometrie und Epidemiologie, LMU München, München, Germany; 5 Medizinische Klinik und Poliklinik I, Klinikum der Universität München, Ziemssenstr. 1, 80336 München, Germany

## Abstract

**Introduction:**

Sepsis is still a leading cause of morbidity and mortality, even in modern times, and thrombocytopenia has been closely associated with unfavorable disease outcome. Decreases in mitochondrial membrane potential (depolarization) were found in different tissues during sepsis. Previous work suggests that mitochondrial dysfunction of platelets correlates with clinical disease activity in sepsis. However, platelet mitochondrial membrane potential (Mmp) has not been investigated in a clinical follow-up design and not with regard to disease outcome.

**Methods:**

In this study, platelet mitochondrial membrane depolarization was assessed by means of a fluorescent Mmp-Index with flow cytometry in 26 patients with sepsis compared with control patients. Platelet Mmp-Index on admission was correlated with the clinical disease scores Acute Physiology and Chronic Health Evaluation Score II (APACHE II), Sequential Organ Failure Score (SOFA), and Simplified Acute Physiology Score II (SAPS II). Finally, platelet Mmp-Index on admission and follow-up were compared in the group of sepsis survivors and nonsurvivors. Expression of the prosurvival protein Bcl-xL in platelets was quantified by immunoblotting.

**Results:**

Platelet mitochondrial membrane depolarization correlated significantly with the simultaneously assessed clinical disease severity by APACHE II (*r* = -0.867; *P* < 0.0001), SOFA (*r* = -0.857; *P* <0.0001), and SAPS II score (*r* = -0.839; *P* < 0.0001). Patients with severe sepsis showed a significant reduction in platelet Mmp-Index compared with sepsis without organ failure (0.18 (0.12 to 0.25) versus 0.79 (0.49 to 0.85), *P* < 0.0006) or with the control group (0.18 (0.12 to 0.25) versus 0.89 (0.68 to 1.00), *P* < 0.0001). Platelet Mmp-Index remained persistently low in sepsis nonsurvivors (0.269 (0.230 to 0.305)), whereas we observed recovery of platelet Mmp-Index in the survivor group (0.9 (0.713 to 1.017)). Furthermore, the level of prosurvival protein Bcl-xL decreased in platelets during severe sepsis.

**Conclusion:**

In this study, we demonstrated that mitochondrial membrane depolarization in platelets correlates with clinical disease severity in patients with sepsis during the disease course and may be a valuable adjunct parameter to aid in the assessment of disease severity, risk stratification, and clinical outcome.

## Introduction

Sepsis is still a leading cause of morbidity and mortality worldwide, despite modern intensive care medicine. Numerous biomarkers have been investigated in the diagnosis and clinical assessment of sepsis, but only few have found their way into clinical routine [1]. Although good clinical scores for disease severity have been developed [2-4], patient assessment on a daily basis remains a challenge and could be facilitated by molecular disease parameters. Evidence is growing that platelets play an active role in fighting infections and in innate immunity [5-8], and thrombocytopenia is frequently observed in systemic infections. Previous studies have found that the severity of thrombocytopenia is associated with increased mortality rates for patients in intensive care units in general [9-13]. Although this has been frequently interpreted as a consumption of platelets [14], signs of cell death have also been observed in platelets. Just lately, our group was able to show that bacteria can directly activate the apoptotic pathway in platelets to induce platelet cell death *in vitro*[15]. Several other groups demonstrated that the septic milieu compromises mitochondrial function, which leads to dysfunction of blood cells, myocardium, and muscle wasting [16-20]. In this context, platelet mitochondrial function was also affected in the sepsis patient, and mitochondrial dysfunction correlated with disease severity and poor outcome in different study designs [21-24]. The focus of this work was to investigate platelet mitochondrial membrane potential (Mmp) with flow cytometry with regard to disease outcome and disease severity in patients with sepsis. Platelet Mmp was correlated with clinical disease-severity scores (APACHE II, SOFA, and SAPS II score) on admission and was compared with that in control patients. We reassessed platelet Mmp in patients with severe sepsis during the disease course and compared it in survivors and nonsurvivors. The protein Bcl-xL, which regulates apoptosis in platelets and is critical for cell survival [25,26], was investigated in severe sepsis with immunoblotting. Overall, in this study, we aimed to investigate whether platelet mitochondrial membrane depolarization could be a potential biomarker of platelets in sepsis. Our hypothesis was that mitochondrial membrane depolarization of platelets correlates with increased disease severity and worse clinical outcome.

## Material and methods

### Study population

Patients with the diagnosis of sepsis or severe sepsis [26], including septic shock as previously defined [27,28] were included in the study between January 2012 and January 2013. Clinical disease severity was assessed with APACHE II, SOFA, and SAPS II scores on the days of blood draws. Severe sepsis was defined as sepsis complicated by organ failure [28]. We considered organ failure as a SOFA point score above 2, as previously used by others [29-31]. Subsequently, the patient collective comprised nine patients with sepsis (nonsevere) and 17 patients with severe sepsis, including septic shock. Patients were recruited from the medical intensive care unit, intermediate care unit, and emergency department of the University Medical Center Innenstadt of the University Hospital Munich. All patients were older than 18 years, and written informed consent was obtained from patients or next of kin. Blood was drawn within the first 48 hours of admission in all patients diagnosed with sepsis and in controls.

In the group of patients with severe sepsis, follow-up blood draws and Mmp reads were taken according to the clinical follow-up assessment of the patient by the critical care team, which was based on organ-failure score SOFA. Follow-up blood draws were initiated if the patient recovered to a SOFA score of less than 3 points or did not show improvement from initial SOFA score after 2 weeks (see Additional file 1). An earlier follow-up blood draw was initiated if the patient showed a rapid clinical deterioration. On average, follow-up reads were taken 7 days after admission. Seventeen control patients with noninfectious medical conditions (including gastrointestinal (GI) bleeding, myocardial infarction, pulmonary embolism, hypertensive urgency, ketoacidosis, arrhythmia, or COPD) were included after written, informed consent (Table 1). Control patients had to have at least two permanent medical conditions requiring medical therapy and were matched by age to the severe-sepsis cohort. For reference, Mmp readings were also taken from healthy volunteers recruited from our staff. Patients with known preexisting or chronic thrombocytopenia or mitochondrial disease, as well as patients with SIRS unrelated to an infectious cause, were excluded from the study. The study was approved by the local ethics committee of the University of Munich in accordance with the Declaration of Helsinki.

**Table 1:** Clinical patient characteristics

	**Sepsis**	**Severe sepsis/septic shock**	**Controls**	
**Number of patients**	9	17	17	
**Male/female**	6/3	9/8	10/7	
**Age**	57 (42 to 71)	69 (55 to 78)	74 (68 to 79)	
**Platelet counts (×10**^**3**^**/μl)**	198 (161 to 210)	98 (79 to 159)	176 (145 to 230)	
**APACHE II score**	9 (8 to 13)	23 (15 to 26)		
**SOFA score**	0 (0 to 1)	7 (5 to 11)		
**SAPS II score**	33 (25 to 35)	53 (40 to 58)		
**Site of infection**			**Admission cause and number of patients**	
Pulmonary	4	7	Cardiac	3
Urinary	0	2	Pulmonary	2
Abdominal	3	5	Endocrine	3
Endocarditis	0	1	Rheumatic	1
Soft tissue	0	1	GI	4
Others	2	1	Other	4

Numbers are given as median and (interquartile range).

### Platelet mitochondrial membrane potential by flow cytometry

Platelets were isolated from whole blood of patients with sepsis and controls, as previously described [15], and mitochondrial membrane potential (Mmp) was measured with JC-1 dye with flow cytometry, according to the manufacturer’s instructions (Immunochemistry Technologies, Bloomington, MN, USA). Then 3 × 10^7^ washed platelets per ml were stained with JC-1 dye for 20 minutes under cell-culture conditions in the dark. Purification and resuspension of platelets in equal volumes and at equal concentrations guaranteed identical staining and processing conditions and equalized the initial differences in platelet number among the clinical samples. Samples from patients with thrombocytopenia were thus fully comparable to samples with normal platelet numbers.

Flow cytometry was performed by using a BD FACSCANTO II flow cytometer (BD Biosciences, Heidelberg, Germany). Mitochondrial membrane potential (Mmp) was assessed as a ratio of the mean FL2 (red fluorescence) and FL1 (green fluorescence), as in previous publications [32]. We called this ratio “Mmp-Index” in the text. A decrease in the FL2-to-FL1 ratio (Mmp-Index) represents a loss in mitochondrial membrane potential (depolarization) (see Additional file 2). Mmp-Index was calculated as the mean of triplicate readings for each patient (see Additional file 1). Stimulation with calcium ionophore A23187 (Calbiochem, Darmstadt, Germany) at 10 μ*M* for 10 minutes, which induces platelet apoptosis and rapid mitochondrial membrane depolarization [33], was used as an internal positive control to assure correct function of the assay in each experiment.

### Protein quantification of Bcl-xL by immunoblotting

Immunoblotting was performed as previously described [15]. In brief, 1 × 10^8^ platelets from patients with sepsis and controls were isolated under identical conditions and lysed in 100 μl of cell-lysis buffer (Cell Signaling Technology, Danvers, MA, USA). Then 30 μg of total protein was transferred into running buffer, and gel electrophoresis was performed according to standard protocols in our laboratory. Protein was blotted onto a PVDF (polyvinylidine difluoride) membrane afterward, and Bcl-xL expression was detected by using a mouse anti-human antibody to Bcl-xL (BD Biosciences, Heidelberg, Germany). β-Actin served as loading control (rabbit anti-human β-Actin; Cell Signaling Technology). Protein expression was quantified by band densitometry of a Bcl-xL-to-actin ratio, which was then compared between groups.

### Statistics

Sample-size calculation for the three groups (sepsis, severe sepsis, and control patients) was calculated on the basis of an estimated 80% difference between groups, the assumption of a 50% standard deviation, a type I error (α-error) < 0.05, and a desired power of 80% (type II error, 20%).

Patient characteristics, clinical scores, and Mmp-Index values are given as median and interquartile range. Correlations of platelet Mmp-Index and clinical disease scores (APACHE II, SOFA, SAPS II) were calculated by using Spearman rank correlation coefficients. Three-group comparisons were based on Kruskal-Wallis tests as global test and *post hoc* Mann–Whitney *U* tests. Admission and follow-up Mmp index values were compared with the Wilcoxon test for paired samples. A *P* value of < 0.05 was considered statistically significant. Because of the exploratory nature of our analyses, we did not adjust the test results for multiple testing. Statistical calculations were performed by using SAS for Unix and Linux, version 9.3 (SAS Institute, Cary, NC, USA).

## Results

### Patient population

In total, 26 patients admitted to our hospital with sepsis, and 17 control patients were included in the study (Table 1). Seventeen patients were subclassified as having severe sepsis including septic shock, whereas nine patients had nonsevere sepsis and were considered to have no organ failure, based on a SOFA score of ≤2. The study population included 15 male and 11 female patients with a median age in the severe-sepsis group of 69 years (55 to 78 years), 57 years (42 to 71 years) in the sepsis group, and 74 years (68 to 79 years) in the control group. No statistical age difference was found among the groups. Site of infection in study patients included pulmonary, urinary, abdominal, soft–tissue, and native heart-valve infections, as shown in Table 1. In the severe-sepsis group, median and interquartile range of APACHE II, SOFA, and SAPS II scores were 23 (15 to 26), 7 (5 to -11) and 53 (40 to 58) and 9 (8 to 13), 0 (0 to 1) and 33 (25 to 35) in the sepsis group (non-severe), respectively. All disease-severity scores were significantly higher in the severe sepsis group compared with the nonsevere sepsis group (APACHE II, *P* = 0.0009; SOFA, *P* = 0.0004 and SAPS II, *P* = 0.0016). Mortality in the severe-sepsis group due to septic organ failure was 41% (7 of 17), whereas only one patient died in the nonsevere sepsis group of a noninfectious cause. Platelet numbers were significantly lower in the severe-sepsis group on admission compared with the nonsevere sepsis and control groups (*P* = 0.003) (Table 1).

### Platelet mitochondrial membrane depolarization correlates with clinical disease severity

**Figure 1:**
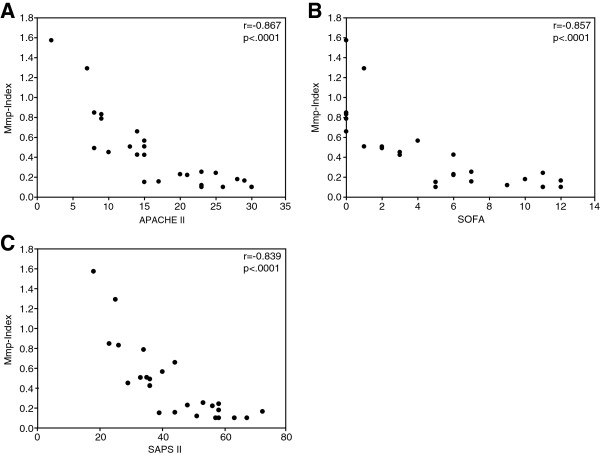
**Correlation of platelet mitochondrial membrane potential with APACHE II, SOFA, and SAPS II scores.** Dot-blot correlation of the individual mean platelet Mmp-Index of each patient calculated from triplicate JC-1 FL2 to FL1 fluorescence ratios by flow cytometry and the clinical disease-severity scores APACHE II **(A)**, SOFA **(B)**, and SAPS II score **(C)** in 26 patients with sepsis. Lower Mmp-Index values indicate a loss of mitochondrial membrane potential, whereas higher clinical disease scores indicate more-severe illness. *r* = statistical correlation coefficient; *P* ≤ 0.05 denotes statistically significant correlation of values.

Mean Mmp-Index of triplicate readings of patients admitted with sepsis (*n* = 26) correlated significantly with the clinical disease-severity scores APACHE II (*r* = -0.867; *P* < 0.0001), SOFA (*r* = -0.857; *P* = 0.0002), and SAPS II (*r* = -0.839; *P* < 0.0001) (Figure 1A-C). A decrease of Mmp-Index that represents a decrease in mitochondrial membrane potential was thus paralleled by higher score numbers, which reflect a more-severe disease status.

### Platelet mitochondrial potential is significantly decreased in patients with severe sepsis

**Figure 2:**
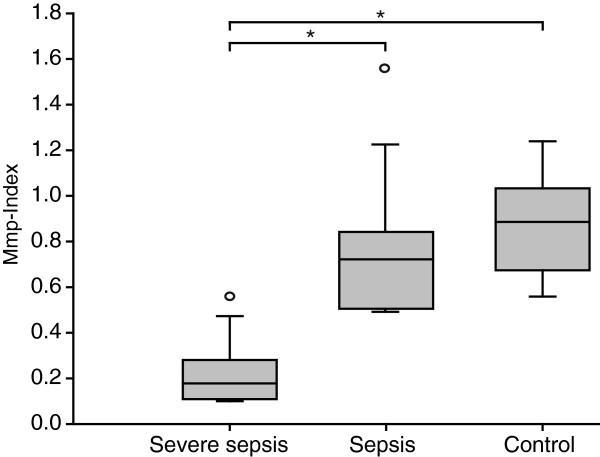
**Comparison of platelet mitochondrial membrane potential of patients with sepsis, severe sepsis, and control patients.** Box-and-whisker plots illustrate platelet Mmp-Index of patients with sepsis (nonsevere without organ failure), severe sepsis including septic shock, and control patients without infection. Lower Mmp-Index values indicate a loss of mitochondrial membrane potential. Platelet Mmp-Index was significantly lower in patients with severe sepsis compared with sepsis without organ failure (*P* < 0.0006) and controls (*P* < 0.0001). No statistical difference was found between patients with sepsis and controls (*P* = 0.42). Box margins identify the upper and lower quartiles; the horizontal line marks the median; and whiskers indicate minimal and maximal values. Outliers are indicated with a circle. *Statistically significant difference, *P* ≤ 0.05.

Platelet Mmp-Index is significantly decreased in patients with severe sepsis, including septic shock, compared with patients with sepsis without organ failure (nonsevere) (0.18 (0.12 to 0.25) versus 0.78 (0.51 to 0.85); *P* < 0.0006) or control patients (Figure 2). No statistically significant differences were noted in platelet Mmp-Indices between the nonsevere sepsis group and control patients (0.78 (0.51 to 0.85) versus 0.89 (0.68 to 1.00); *P* = 0.42). Among the sepsis patients, APACHE II, SOFA, and SAPS II scores were significantly higher in the severe-sepsis subgroup compared with the non-severe sepsis population. For reference, healthy volunteers (*n* = 14) showed platelet Mmp index values of 1.096 (1.049 to 1.398).

### Platelet mitochondrial membrane depolarization correlates with clinical disease outcome

**Figure 3:**
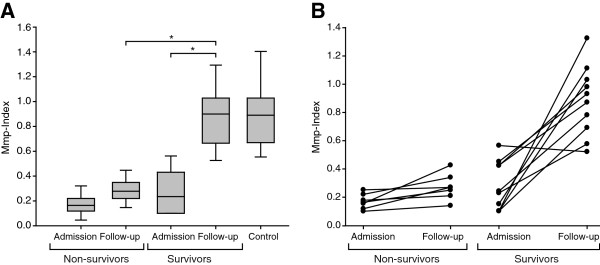
**Comparison of platelet mitochondrial membrane potential of survivors and nonsurvivors of the severe-sepsis group. (A)** Box-and whisker plots illustrate platelet Mmp-Index of survivors (*n* = 10) and nonsurvivors (*n* = 7) of the severe-sepsis group on admission and during follow-up. Lower Mmp-Index values indicate a loss of mitochondrial membrane potential. Platelet Mmp-Index did not show significant differences between the groups on admission (*P* = 0.44). No relevant increase was noted in platelet Mmp-Index in nonsurvivors during follow-up (Mmp-Index <0.5), whereas Mmp-Index in survivors recovered to baseline levels of controls. Platelet Mmp-Index was significantly higher in survivors than in nonsurvivors on follow-up (*P* = 0.004). Box margins identify the upper and lower quartile; the horizontal line marks the median; and whiskers indicate minimal and maximal values. Outliers are indicated with a circle. *Statistically significant difference, *P* ≤ 0.05. **(B)** Individual mean Mmp-Index values from triplicate readings of patients in the group of sepsis survivors and nonsurvivors on admission and during follow-up.

Median platelet Mmp-Index of the group of patients with severe sepsis who died as a consequence of sepsis was compared with that of sepsis survivors. On admission, no significant difference occurred in platelet mitochondrial membrane potential between survivors and nonsurvivors, with a trend to less Mmp-depolarization in nonsurvivors (*P* = 0.43) (Figure 3A). Median APACHE II, SOFA, and SAPS II scores also did not differ between the groups of survivors and nonsurvivors on admission (*P* = 0.14; *P* = 0.14; and *P* = 0.18). During the clinical course, significant recovery of the Mmp-Index was observed in the group of survivors (0.235 (0.113 to 0.424) to 0.9 (0.713 to 1.017)), whereas persistently low platelet Mmp values (<0.5 Mmp-Index value) were recorded in the nonsurvivors group (Figure 3A). Platelet Mmp-Index on follow-up subsequently showed a significant difference between the group of sepsis survivors and nonsurvivors on follow-up (0.269 (0.230 to 0.305) versus 0.9 (0.713 to 1.017)). No significant difference was found in the follow-up platelet Mmp-Index of survivors and control patients. Illustration of individual mean Mmp values for each patient demonstrates the lack of recovery in nonsurvivors of the severe-sepsis group, which all remained below 0.5 Mmp-Index, and the recovery of Mmp-Index values in survivors on follow-up (Figure 3B and Additional file 1).

Follow-up SOFA scores of nonsurvivors had not improved at 2 weeks’ assessment, whereas SOFA scores in survivors improved significantly. Admission and follow-up SOFA scores, corresponding mean Mmp values with SEM from triplicate readings for each patient, and the day of the follow-up blood draw in relation to the patient’s day of discharge or the day a patient died are shown in Additional file 1.

### Bcl-xL expression is decreased in platelets during severe sepsis

**Figure 4:**
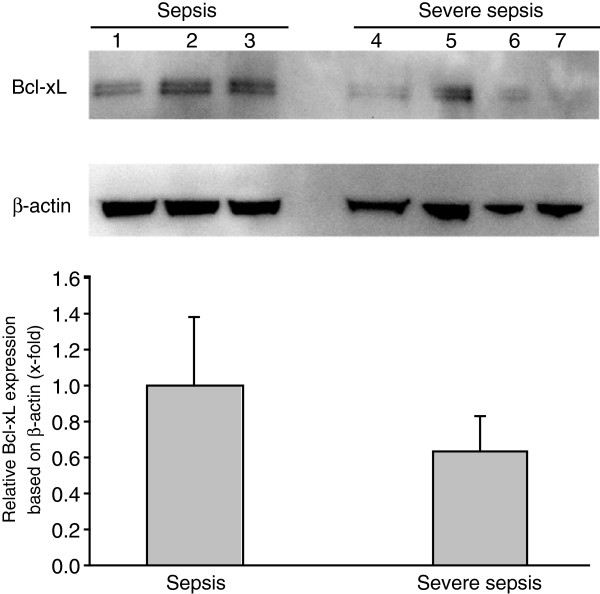
**Comparison of Bcl-xL expression in platelets of patients with sepsis and severe sepsis.** Platelet Bcl-xL expression in patients with sepsis (lanes 1 to 3) and severe sepsis (lanes 4 to 7) on day 1 of admission was compared with immunoblotting. Bcl-xL expression was quantified with band-densitometry analysis with the help of a Bcl-xL-to-actin ratio. Mean Bcl-xL-to-actin ratio for the sepsis group was set as 1, and relative decrease in Bcl-xL expression in the severe-sepsis group was compared accordingly. Relative Bcl-xL expression between groups based on actin loading is shown.

Bcl-xL expression in platelets was demonstrated on admission in three sepsis patients with an Mmp-Index above 0.8 and nonsevere sepsis compared with four patients with severe sepsis and low Mmp-Index (Figure 4). Samples of all these patients were taken on day 1 of admission. Bcl-xL expression was decreased in platelets during severe sepsis compared with the sepsis group. Bcl-xL protein expression was quantified with band densitometry based on actin loading. Mean Bcl-xL-to-actin ratio of the sepsis group was set as 1, and severe-sepsis patients were compared by ratio accordingly. Decrease in Bcl-xL expression in the severe sepsis group is shown as relative Bcl-xL expression based on actin.

## Discussion

In this study, we demonstrated that platelet mitochondrial membrane depolarization correlates with disease severity (APACHE II, SOFA, and SAPS II score) and disease outcome in patients with sepsis. Mmp-Index was significantly decreased in a subgroup of patients with severe sepsis compared with sepsis patients without organ failure (nonsevere) and controls (Figure 2). Platelet counts in the severe-sepsis group were significantly reduced compared with the other groups, which implies that the increased apoptotic activity in the septic milieu reflects the loss of platelets and may explain the prognostic relevance of thrombocytopenia. Patients classified as sepsis without organ failure (nonsevere) showed no significant loss of mitochondrial membrane potential compared with control patients. This is likely because predicted mortality based on APACHE II, SOFA, and SAPS II scores in our patients with nonsevere sepsis was relatively low.

Overall, the link of thrombocytopenia as a predictor of clinical prognosis in patients in the ICU has been well established, although investigations on a molecular level are still rare. Besides a loss of platelets, hemostatic functions of platelets, such as aggregation, are impaired during sepsis, which also correlates with the severity of infection [34]. During sepsis, platelets release prothrombotic particles, which can activate systemic coagulation and lead to lethal embolic events [35]. Growing evidence suggests that platelets have the capacity to fight bacterial infections directly, and that they act as cells of innate immunity [5,7,8]. We and others have been able to demonstrate that platelets directly interact with bacteria and release bactericidal proteins that inhibit bacterial growth [6,36-38]. Bacteria in return release potent exotoxins that drive platelets into cell death [15]. It is thus not surprising that platelet numbers decline at a rate that reflects infectious disease activity and that thrombocytopenia has prognostic value, as suggested by previous work [10]. Therefore, it is tempting to speculate that the loss of platelets is a result of direct bacterial action or the septic milieu rather than random clearance and consumption of platelets.

Mitochondria are the central target in the intrinsic apoptotic pathway leading to platelet apoptosis [25], and mitochondrial damage constitutes an early indicator of cell death in platelets [33]. Although closely linked to apoptosis [25,33,39,40], mitochondrial dysfunction is not an exclusive sign of cell death and can also occur with microcirculatory distress [41,42]. During clinical or experimental sepsis, mitochondrial dysfunction has also been observed in heart and skeletal muscle tissue [18,19], as well as in apoptosis of blood cells such as monocytes [20] and lymphocytes [43]. Based on experimental data from our own group and observations by others, we have reason to believe that mitochondrial dysfunction, platelet cell death, and thrombocytopenia are not coincidental in the septic milieu. We have previously shown that live bacteria (*E*scherichia *coli, S*taphylococcus *aureus*) isolated from sepsis directly activate a calpain-dependent degradation pathway that eliminates the anti-apoptotic protein Bcl-xL [15]. Bcl-xL is critical for platelet survival, and inhibition or neutralization of Bcl-xL induces platelet cell death. This process results in platelet mitochondrial membrane depolarization and programmed cell death of platelets *in vitro*. We measured Bcl-xL protein expression in platelets from patients with sepsis on admission (day 1) and found decreased expression of Bcl-xL in patients with severe sepsis who had low Mmp-Index values compared with patients with nonsevere sepsis (Figure 4) in this study. We concluded that the septic milieu probably generates a multitude of factors, including toxins that damage platelets and reflect the degree of septic activity.

Other groups have described that bacterial factors of *S. aureus* induce mitochondrial membrane depolarization and apoptosis in platelets [44]. Although these observations support the hypothesis that mitochondrial dysfunction, apoptosis and thrombocytopenia are physiologically connected, we cannot prove the link in this complex *in vivo* system, but they appear to be reasonable explanations of factors that contribute to the process of sepsis-induced thrombocytopenia. Undoubtedly, the etiology of thrombocytopenia in sepsis is multifactorial and includes microcirculatory and coagulation-associated effects.

A previous study first demonstrated a correlation of platelet mitochondrial membrane depolarization and clinical disease severity on admission in a diverse group of medical and surgical ICU patients with SIRS by a different approach [24]. Before that, Sjövall and colleagues [23] observed a substantial increase in platelet mitochondrial respiratory capacity in nonsurvivors compared with survivors of sepsis and demonstrated an association between mitochondrial dysfunction and clinical outcome in sepsis. They further detected mitochondrial damage by release of cytochrome *c* from mitochondria. However, no correlation between mitochondrial respiration and standard clinical assessment scores for illness (APACHE II, SAPS, and SOFA Score) was found. Most likely, this is explained by a different approach to assess mitochondrial dysfunction, which probably differs from the loss of mitochondrial membrane potential in the apoptotic process.

In this study, we further aimed to investigate whether the degree of platelet mitochondrial membrane depolarization could also be correlated with disease severity during the disease course and outcome. We compared platelet mitochondrial membrane depolarization in a subgroup of patients with severe sepsis, including septic shock on admission and during clinical disease course, and compared Mmp-Index in survivors and nonsurvivors. On admission, initial Mmp-Index values in the severe-sepsis group were significantly decreased compared with controls, but no significant difference between survivors and nonsurvivors on admission was found (Figure 3A). Supporting these findings, no difference was noted regarding the clinical disease-severity scores APACHE II, SOFA, and SAPS II on admission between survivors and nonsurvivors in this study. Individual and group analysis of patients from the sepsis group illustrates that patients who survived sepsis showed recovery of Mmp-Index on follow-up, whereas no significant recovery in sepsis nonsurvivors was observed (Mmp-Index <0.5) (Figure 3A, B, and Additional file 1). It appears that follow-up Mmp-reads to identify clinical trends may be equally important to the absolute Mmp-values. Mmp-Index may prove a valuable tool to evaluate clinical recovery of the individual patient in addition to clinical parameters. The data suggest that markers of platelet apoptosis, such as mitochondrial membrane potential and Bcl-xL levels, may reflect infectious disease activity and disease severity in sepsis.

## Conclusions

Platelet Mmp-Index proved to be a highly reproducible, easily accessible readout for mitochondrial integrity and showed good correlation with clinical disease severity during clinical course, correlated with clinical outcome, and normalized with recovery. In addition, we detected a proapoptotic phenotype of platelets based on Bcl-xL levels in patients with severe sepsis compared with controls. Further research on larger patient populations will, however, be necessary to establish markers of platelet apoptosis in daily routine. Nonetheless, results from this study and previous investigations seem to promise that markers of platelet apoptosis may assist in the assessment of disease severity, in risk stratification, and in management of patients with sepsis in the future.

## Key messages

• Platelet mitochondrial membrane depolarization and decrease of prosurvival proteins correlate with clinical disease severity in patients with sepsis.

• Platelet mitochondrial membrane potential correlates with disease outcome, remains low with unfavorable disease progression, and normalizes with clinical recovery.

• Markers of platelet apoptosis may aid in the assessment and risk stratification of patients with sepsis.

## Abbreviations

APACHE II: Acute physiology and chronic health evaluation score II; COPD: chronic obstructive pulmonary disease; GI: gastrointestinal; Mmp: mitochondrial membrane potential; SAPS II: Simplified Acute Physiology Score II; SIRS: systemic inflammatory response syndrome; SOFA: Sequential Organ Failure Score (SOFA).

## Competing interests

The authors declare that they have no competing interests.

## Authors’ contributions

KG, experimental design, data collection and analysis, figure preparation, final approval of the manuscript; MA, patient enrollment, manuscript writing, critical revision, and final approval of the manuscript; RH, patient enrollment, data collection and analysis, final approval of the manuscript; PB, patient enrollment, data collection and analysis, final approval of the manuscript; TA, conception and design, critical revision, and final approval of the manuscript; AC, statistics and calculations, data analysis, final approval of the manuscript; HYS, conception and design, manuscript writing, final approval of the manuscript; SM, conception and design, critical revision, and final approval of the manuscript; BFK, conception and design, data collection and analysis, manuscript writing, and final approval of the manuscript. All authors read and approved the final manuscript.

## Supplementary Material

Additional file 1 **Individual outcome and characteristics of patients with severe sepsis.** The table summarizes the individual SOFA scores on admission and follow-up, the corresponding individual mean Mmp values from triplicate readings with SEM for each patient and the day follow-up blood was drawn in relation to the day of a patient’s discharge (DC) in survivors, and the day a patient died (D) in nonsurvivors of the severe-sepsis group. (a) admission, (f) follow-up; DC, day of discharge; D, day of death. Click here for file

Additional file 2 **Illustration of Mmp-Index calculation with flow cytometry.** Flow-cytometry analysis of platelets from a patient with sepsis (A) and a patient with severe sepsis (B) stained with JC-1 are shown. Mitochondrial membrane depolarization is characterized by a decrease in mean red fluorescence (y-axis, FL2) and an increase in mean green fluorescence (x-axis, FL1) visible in the severe-sepsis sample. Fluorescence means of red (FL2)-fluorescence and green (FL1)-fluorescence of the platelet population (circle) are measured, and the ratio FL2 (red) divided by FL1 (green)-fluorescence generates the Mmp-Index. A lower Mmp index thus represents a loss in mitochondrial membrane potential. Click here for file

## References

[B1] Pierrakos C Vincent JL Sepsis biomarkers: a review Crit Care 2010 14 R15 10.1186/cc8872 20144219 PMC2875530

[B2] Knaus WA Draper EA Wagner DP Zimmerman JE APACHE II: a severity of disease classification system Crit Care Med 1985 13 818 829 10.1097/00003246-198510000-00009 3928249

[B3] Le Gall JR Lemeshow S Saulnier F A new Simplified Acute Physiology Score (SAPS II) based on a European/North American multicenter study JAMA 1993 270 2957 2963 10.1001/jama.1993.03510240069035 8254858

[B4] Vincent JL de Mendonca A Cantraine F Moreno R Takala J Suter PM Sprung CL Colardyn F Blecher S Use of the SOFA score to assess the incidence of organ dysfunction/failure in intensive care units: results of a multicenter, prospective study: Working Group on “sepsis-related problems” of the European Society of Intensive Care Medicine Crit Care Med 1998 26 1793 1800 10.1097/00003246-199811000-00016 9824069

[B5] Engelmann B Massberg S Thrombosis as an intravascular effector of innate immunity Nat Rev Immunol 2013 13 34 45 23222502 10.1038/nri3345

[B6] Kraemer BF Campbell RA Schwertz H Cody MJ Franks Z Tolley ND Kahr WH Lindemann S Seizer P Yost CC Zimmerman GA Weyrich AS Novel anti-bacterial activities of beta-defense 1 in human platelets: suppression of pathogen growth and signaling of neutrophil extracellular trap formation PLoS Pathog 2011 7 e1002355 10.1371/journal.ppat.1002355 22102811 PMC3213094

[B7] Semple JW Freedman J Platelets and innate immunity Cell Mol Life Sci 2010 67 499 511 10.1007/s00018-009-0205-1 20016997 PMC11115613

[B8] Weyrich AS Zimmerman GA Platelets: signaling cells in the immune continuum Trends Immunol 2004 25 489 495 10.1016/j.it.2004.07.003 15324742

[B9] Gafter-Gvili A Mansur N Bivas A Zemer-Wassercug N Bishara J Leibovici L Paul M Thrombocytopenia in *Staphylococcus aureus* bacteremia: risk factors and prognostic importance Mayo Clin Proc 2011 86 389 396 10.4065/mcp.2010.0705 21531882 PMC3084641

[B10] Vandijck DM Blot SI De Waele JJ Hoste EA Vandewoude KH Decruyenaere JM Thrombocytopenia and outcome in critically ill patients with bloodstream infection Heart Lung 2010 39 21 26 10.1016/j.hrtlng.2009.07.005 20109983

[B11] Akca S Haji-Michael P de Mendonca A Suter P Levi M Vincent JL Time course of platelet counts in critically ill patients Crit Care Med 2002 30 753 756 10.1097/00003246-200204000-00005 11940740

[B12] Strauss R Wehler M Mehler K Kreutzer D Koebnick C Hahn EG Thrombocytopenia in patients in the medical intensive care unit: bleeding prevalence, transfusion requirements, and outcome Crit Care Med 2002 30 1765 1771 10.1097/00003246-200208000-00015 12163790

[B13] Couto-Alves A Wright VJ Perumal K Binder A Carrol ED Emonts M de Groot R Hazelzet J Kuijpers T Nadel S Zenz W Ramnarayan P Levin M Coin L Inwald DP A new scoring system derived from base excess and platelet count at presentation predicts mortality in paediatric meningococcal sepsis Crit Care 2013 17 R68 10.1186/cc12609 23577792 PMC3672696

[B14] Bozza FA Shah AM Weyrich AS Zimmerman GA Amicus or adversary: platelets in lung biology, acute injury, and inflammation Am J Respir Cell Mol Biol 2009 40 123 134 10.1165/rcmb.2008-0241TR 18723438 PMC2633137

[B15] Kraemer BF Campbell RA Schwertz H Franks ZG Vieira de Abreu A Grundler K Kile BT Dhakal BK Rondina MT Kahr WH Mulvey MA Blaylock RC Zimmerman GA Weyrich AS Bacteria differentially induce degradation of Bcl-xL, a survival protein, by human platelets Blood 2012 120 5014 5020 10.1182/blood-2012-04-420661 23086749 PMC3525025

[B16] Garrabou G Moren C Lopez S Tobias E Cardellach F Miro O Casademont J The effects of sepsis on mitochondria J Infect Dis 2012 205 392 400 10.1093/infdis/jir764 22180620

[B17] Harrois A Huet O Duranteau J Alterations of mitochondrial function in sepsis and critical illness Curr Opin Anaesthesiol 2009 22 143 149 10.1097/ACO.0b013e328328d1cc 19390243

[B18] Brealey D Brand M Hargreaves I Heales S Land J Smolenski R Davies NA Cooper CE Singer M Association between mitochondrial dysfunction and severity and outcome of septic shock Lancet 2002 360 219 223 10.1016/S0140-6736(02)09459-X 12133657

[B19] Rudiger A Singer M Mechanisms of sepsis-induced cardiac dysfunction Crit Care Med 2007 35 1599 1608 10.1097/01.CCM.0000266683.64081.02 17452940

[B20] Adrie C Bachelet M Vayssier-Taussat M Russo-Marie F Bouchaert I Adib-Conquy M Cavaillon JM Pinsky MR Dhainaut JF Polla BS Mitochondrial membrane potential and apoptosis peripheral blood monocytes in severe human sepsis Am J Respir Crit Care Med 2001 164 389 395 10.1164/ajrccm.164.3.2009088 11500338

[B21] Lorente L Iceta R Martin MM Lopez-Gallardo E Sole-Violan J Blanquer J Labarta L Diaz C Jimenez A Montoya J Ruiz-Pesini E Survival and mitochondrial function in septic patients according to mitochondrial DNA haplogroup Crit Care 2012 16 R10 10.1186/cc11150 22251664 PMC3396241

[B22] Lorente L Martin MM Lopez-Gallardo E Iceta R Sole-Violan J Blanquer J Labarta L Diaz C Jimenez A Lafuente N Hernandez M Mendez F Medina N Ferrer-Agüero JM Ferreres J Lliminana MC Mora ML Lubillo S Sanchez-Palacios M Montoya J Ruiz-Pedini E Platelet cytochrome c oxidase activity and quantity in septic patients Crit Care Med 2011 39 1289 1294 10.1097/CCM.0b013e31820ee20c 21297457

[B23] Sjovall F Morota S Hansson MJ Friberg H Gnaiger E Elmer E Temporal increase of platelet mitochondrial respiration is negatively associated with clinical outcome in patients with sepsis Crit Care 2010 14 R214 10.1186/cc9337 21106065 PMC3219983

[B24] Yamakawa K Ogura H Koh T Ogawa Y Matsumoto N Kuwagata Y Shimazu T Platelet mitochondrial membrane potential correlates with severity in patients with systemic inflammatory response syndrome J Trauma Acute Care Surg 2013 74 411 417 discussion 418 10.1097/TA.0b013e31827a34cf 23354232

[B25] Kile BT The role of the intrinsic apoptosis pathway in platelet life and death J Thromb Haemost 2009 7 214 217 19630803 10.1111/j.1538-7836.2009.03366.x

[B26] Mason KD Carpinelli MR Fletcher JI Collinge JE Hilton AA Ellis S Kelly PN Ekert PG Metcalf D Roberts AW Huang DC Kile BT Programmed anuclear cell death delimits platelet life span Cell 2007 128 1173 1186 10.1016/j.cell.2007.01.037 17382885

[B27] Bone RC Balk RA Cerra FB Dellinger RP Fein AM Knaus WA Schein RM Sibbald WJ Definitions for sepsis and organ failure and guidelines for the use of innovative therapies in sepsis: The ACCP/SCCM Consensus Conference Committee, American College of Chest Physicians/Society of Critical Care Medicine Chest 1992 101 1644 1655 10.1378/chest.101.6.1644 1303622

[B28] Levy MM Fink MP Marshall JC Abraham E Angus D Cook D Cohen J Opal SM Vincent JL Ramsay G 2001 SCCM/ESICM/ACCP/ATS/SIS International Sepsis Definitions Conference Crit Care Med 2003 31 1250 1256 10.1097/01.CCM.0000050454.01978.3B 12682500

[B29] Ceriani R Mazzoni M Bortone F Gandini S Solinas C Susini G Parodi O Application of the sequential organ failure assessment score to cardiac surgical patients Chest 2003 123 1229 1239 10.1378/chest.123.4.1229 12684316

[B30] Ulvik A Kvale R Wentzel-Larsen T Flaatten H Multiple organ failure after trauma affects even long-term survival and functional status Crit Care 2007 11 R95 10.1186/cc6111 17784940 PMC2556737

[B31] Vincent JL Moreno R Takala J Willatts S De Mendonca A Bruining H Reinhart CK Suter PM Thijs LG The SOFA (Sepsis-related Organ Failure Assessment) score to describe organ dysfunction/failure, on behalf of the Working Group on Sepsis-Related Problems of the European Society of Intensive Care Medicine Intensive Care Med 1996 22 707 710 10.1007/BF01709751 8844239

[B32] Verhoeven AJ Verhaar R Gouwerok EG de Korte D The mitochondrial membrane potential in human platelets: a sensitive parameter for platelet quality Transfusion 2005 45 82 89 10.1111/j.1537-2995.2005.04023.x 15647022

[B33] Leytin V Allen DJ Mutlu A Gyulkhandanyan AV Mykhaylov S Freedman J Mitochondrial control of platelet apoptosis: effect of cyclosporin A, an inhibitor of the mitochondrial permeability transition pore Lab Invest 2009 89 374 384 10.1038/labinvest.2009.13 19238135

[B34] Yaguchi A Lobo FL Vincent JL Pradier O Platelet function in sepsis J Thromb Haemost 2004 2 2096 2102 10.1111/j.1538-7836.2004.01009.x 15613012

[B35] Azevedo LC Janiszewski M Pontieri V de Pedro M A Bassi E Tucci PJ Laurindo FR Platelet-derived exosomes from septic shock patients induce myocardial dysfunction Crit Care 2007 11 R120 10.1186/cc6176 17996049 PMC2246209

[B36] Rondina MT Schwertz H Harris ES Kraemer BF Campbell RA Mackman N Grissom CK Weyrich AS Zimmerman GA The septic milieu triggers expression of spliced tissue factor mRNA in human platelets J Thromb Haemost 2011 9 748 758 10.1111/j.1538-7836.2011.04208.x 21255247 PMC3071458

[B37] Yeaman MR Bayer AS Koo SP Foss W Sullam PM Platelet microbicidal proteins and neutrophil defensin disrupt the *Staphylococcus aureus* cytoplasmic membrane by distinct mechanisms of action J Clin Invest 1998 101 178 187 10.1172/JCI562 9421480 PMC508554

[B38] Yeaman MR Sullam PM Dazin PF Bayer AS Platelet microbicidal protein alone and in combination with antibiotics reduces *Staphylococcus aureus* adherence to platelets in vitro Infect Immun 1994 62 3416 3423 8039912 10.1128/iai.62.8.3416-3423.1994 PMC302973

[B39] Kroemer G Mitochondrial control of apoptosis: an introduction Biochem Biophys Res Commun 2003 304 433 435 10.1016/S0006-291X(03)00614-4 12729576

[B40] Zamzami N Marchetti P Castedo M Zanin C Vayssiere JL Petit PX Kroemer G Reduction in mitochondrial potential constitutes an early irreversible step of programmed lymphocyte death in vivo J Exp Med 1995 181 1661 1672 10.1084/jem.181.5.1661 7722446 PMC2192017

[B41] Fink MP Cytopathic hypoxia: mitochondrial dysfunction as mechanism contributing to organ dysfunction in sepsis Crit Care Clin 2001 17 219 237 10.1016/S0749-0704(05)70161-5 11219231

[B42] Ince C The microcirculation is the motor of sepsis Crit Care 2005 9 S13 S19 10.1186/cc3753 16168069 PMC3226164

[B43] Hotchkiss RS Swanson PE Knudson CM Chang KC Cobb JP Osborne DF Zollner KM Buchman TG Korsmeyer SJ Karl IE Overexpression of Bcl-2 in transgenic mice decreases apoptosis and improves survival in sepsis J Immunol 1999 162 4148 4156 10201940

[B44] Towhid ST Nega M Schmidt EM Schmid E Albrecht T Munzer P Borst O Gotz F Lang F Stimulation of platelet apoptosis by peptidoglycan from *Staphylococcus aureus* 113 Apoptosis 2012 17 998 1008 10.1007/s10495-012-0718-1 22752708

