# Supplementary material for: Platelet mitochondrial membrane depolarization reflects disease severity in patients with sepsis and correlates with clinical outcome
Source: Crit Care. 2014 Feb 12;18(1):R31. doi: 10.1186/cc13724 (PMC4056796; doi:10.1186/cc13724)
Supplement: Additional file 1 — Individual outcome and characteristics of patients with severe sepsis. The table summarizes the individual SOFA scores on admission and follow-up, the corresponding individual mean Mmp values from triplicate readings with SEM for each patient and the day follow-up blood was drawn in relation to the day of a patient’s discharge (DC) in survivors, and the day a patient died (D) in nonsurvivors of the severe-sepsis group. (a) admission, (f) follow-up; DC, day of discharge; D, day of death. [file cc13724-S1.pdf]

Survivor/Non-survivor	SOFA (a)	SOFA (f)	Mmp (a)	Mmp (f)	Day (f)	Day DC/D
Non-survivor	14	11	0.164±0.015	0.251±0.023	14	30
Non-survivor	6	9	0.224±0.017	0.340±0.030	9	14
Non-survivor	11	10	0.112±0.007	0.126±0.012	3	5
Non-survivor	10	11	0.178±0.012	0.211±0.021	4	7
Non-survivor	8	7	0.155±0.003	0.426±0.025	13	17
Non-survivor	9	10	0.118±0.012	0.270±0.014	14	28
Non-survivor	7	9	0.252±0.017	0.269±0.019	14	21
Survivor	12	2	0.110±0.009	0.690±0.053	13	21
Survivor	4	1	0.564±0.006	0.524±0.034	4	6
Survivor	6	1	0.424±0.038	0.870±0.038	3	6
Survivor	5	1	0.125±0.007	1.324±0.093	3	3
Survivor	3	0	0.422±0.018	0.979±0.042	3	4
Survivor	5	2	0.228±0.009	0.575±0.045	5	8
Survivor	13	1	0.120±0.010	1.032±0.085	11	18
Survivor	3	0	0.457±0.023	0.935±0.062	4	5
Survivor	13	2	0.241±0.005	0.786±0.037	7	11
Survivor	5	0	0.152±0.014	1.111±0.055	3	6
