# Supplementary figures and images for: Platelet mitochondrial membrane depolarization reflects disease severity in patients with sepsis and correlates with clinical outcome
Source: Crit Care. 2014 Feb 12;18(1):R31. doi: 10.1186/cc13724 (PMC4056796; doi:10.1186/cc13724)

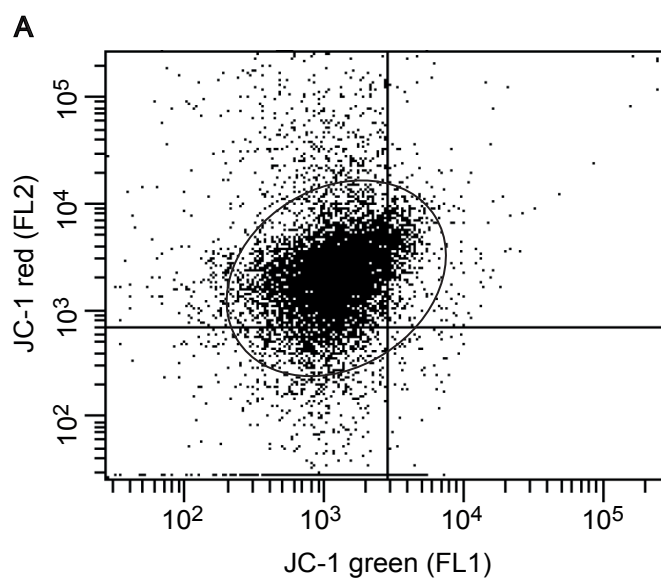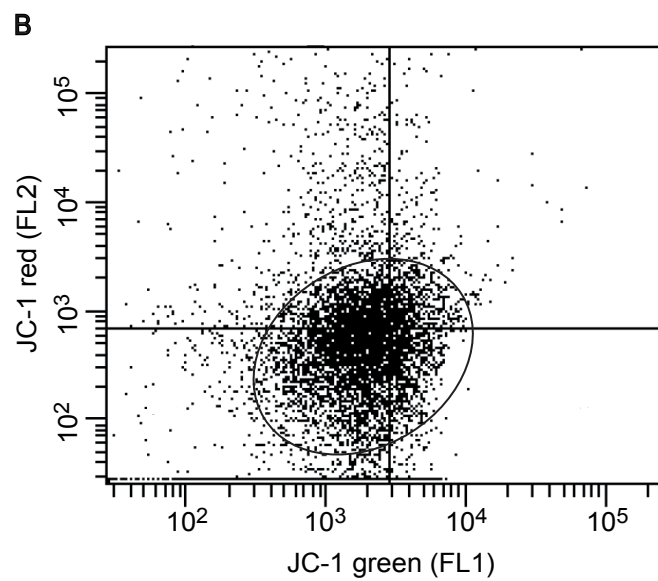

Gründler *et al.*\_supplemental figure 1

Supplement: Additional file 2 — Illustration of Mmp-Index calculation with flow cytometry. Flow-cytometry analysis of platelets from a patient with sepsis (A) and a patient with severe sepsis (B) stained with JC-1 are shown. Mitochondrial membrane depolarization is characterized by a decrease in mean red fluorescence (y-axis, FL2) and an increase in mean green fluorescence (x-axis, FL1) visible in the severe-sepsis sample. Fluorescence means of red (FL2)-fluorescence and green (FL1)-fluorescence of the platelet population (circle) are measured, and the ratio FL2 (red) divided by FL1 (green)-fluorescence generates the Mmp-Index. A lower Mmp index thus represents a loss in mitochondrial membrane potential. [file cc13724-S2.pdf]
